# The Geriatric Nutritional Risk Index predicts postoperative complications and prognosis in elderly patients with colorectal cancer after curative surgery

**DOI:** 10.1038/s41598-020-67285-y

**Published:** 2020-07-01

**Authors:** Masaru Sasaki, Norikatsu Miyoshi, Shiki Fujino, Takayuki Ogino, Hidekazu Takahashi, Mamoru Uemura, Chu Matsuda, Hirofumi Yamamoto, Tsunekazu Mizushima, Masaki Mori, Yuichiro Doki

**Affiliations:** 1 Department of Gastroenterological Surgery, Osaka University Graduate School of Medicine, Suita, Japan; 2 Department of Innovative Oncology Research and Regenerative Medicine, Osaka International Cancer Institute, Osaka, Japan; 3 Department of Surgery and Science, Graduate School of Medical Sciences, Kyushu University, Fukuoka, Japan

**Keywords:** Colorectal cancer, Risk factors, Prognostic markers, Outcomes research, Surgical oncology

## Abstract

Malnutrition has been considered to be associated with the prognosis of cancer. The Geriatric Nutritional Risk Index (GNRI), based on serum albumin levels, present body weight, and ideal body weight, is a simple screening tool to predict the risk of nutrition-related morbidity and mortality in elderly patients. We aimed to evaluate whether preoperative GNRI was associated with postoperative complications and prognosis in elderly patients with colorectal cancer (CRC). We retrospectively enrolled 313 CRC patients aged ≥65 years after curative surgery and classified them into an all-risk GNRI (≤98) group and a no-risk GNRI (>98) group. Kaplan-Meier analysis showed overall survival was significantly worse in the all-risk GNRI group than in the no-risk GNRI group (*P* = 0.009). Multivariable analyses showed low GNRI (≤98) was an independent risk factor for postoperative complications (*P* = 0.048) and overall survival (*P* = 0.001) in the patients. Among the complications, the incidence of surgical site infection, in particular, was significantly higher in the all-risk GNRI group (*P* = 0.008). In conclusion, low preoperative GNRI (≤98) was associated with increased postoperative complications and poor prognosis. Preoperative GNRI can be used as an identifier for potential high-risk group of morbidity and mortality in elderly CRC patients.

## Introduction

Colorectal cancer (CRC) is the third most commonly diagnosed cancer and the second leading cause of cancer-related mortality worldwide^1,2^. According to the World Health Organization GLOBOCAN database, there were an estimated 1,849,518 new CRC cases and 880,792 CRC-related deaths in 2018^3^. As life expectancy increases and the population ages, the number of elderly patients undergoing surgery also increases^4,5^. For instance, in the United States, 60.7% of all the incident CRC patients in 2018 were 65 years or older, and then 81% of the elderly patients and even 64% of the patients aged ≥85 years underwent surgery from 2011 to 2015^3,6^.

Elderly patients often have some comorbidities, such as cardiovascular disease and respiratory dysfunction^7,8^, and often become malnourished^9,10^. In elderly patients, disease-related malnutrition is associated with increased morbidity and mortality^9–12^ and prolonged length of stay in hospital due to decrease in their life activity, performance status, and immune function^11–14^.

The Geriatric Nutritional Risk Index (GNRI) is an elderly-specific index that has been proposed to assess the nutrition-related risk of morbidity and mortality for elderly patients in hospital^15,16^. This index was first reported by Bouillanne *et al*. They divided patients into four groups—a major-risk group (GNRI: <82), a moderate-risk group (GNRI: 82–<92), a low-risk group (GNRI: 92–98), and a no-risk group (GNRI: >98)—and suggested that the risk of infectious complications or mortality was significantly higher in the major-, moderate-, and low-risk groups than in the no-risk group^17^. The GNRI is also used for prognosis of chronic diseases^18–20^, and in recent years, it has been reported as a useful screening tool to predict prognosis for not only chronic diseases but also malignant tumors^21–24^.

To date, there have been no reports on the relationship between GNRI and short- or long-term outcomes for elderly patients with CRC after surgery. Therefore, in this study, we investigated whether preoperative GNRI was associated with postoperative complications and prognosis for elderly patients with CRC who underwent curative surgery.

## Methods

### Patients and datasets

This study retrospectively enrolled 313 patients with CRC aged ≥65 years who underwent curative resection at Osaka University Hospital from August 2007 to December 2012. Patients who underwent curative resection for distant metastases were also included. Exclusion criteria for patients were as follows: (1) aged <65 years, (2) surgery for recurrence, (3) multiple primaries, (4) colitic cancer, (5) received neoadjuvant chemotherapy, (6) underwent transanal endoscopic microsurgery, (7) cases which lacked any of preoperative laboratory data or pathological findings described in Table 1. Two hundred and eighteen elderly CRC patients who underwent curative surgery at Osaka International Cancer Institute from January 2007 to December 2013 were enrolled according to the same criteria as described above, and analyzed as another dataset.

**Table 1:** The characteristics of 313 patients with CRC.

Variables	Total (n = 313)
Age (years)*	73 (65–94)
Sex (male/female)	201/112
BMI (kg/m^2^)*	22.2 (8.7–33.6)
ALB (g/dL)*	3.8 (1.9–4.8)
WBC (/μL)*	5610 (2360–13700)
CRP (mg/dl)*	0.07 (0.04–9.07)
Preoperative CEA (ng/mL)*	3 (0.1–321)
Preoperative CA19–9 (U/mL)*	11 (0–2505)
Tumor location (colon/rectum)	239/74
Degree of differentiation (tub1/tub2/por/pap/muc)	132/156/14/1/10
Depth of tumor invasion (Tis/T1/T2/T3/T4)	29/73/58/136/17
Lymph node metastasis (N0/N1/N2)	225/65/23
Lymphatic vessel invasion (ly0/ly1/ly2/ly3)	117/163/29/4
Venous invasion (v0/v1/v2/v3)	238/62/12/1
Distant metastasis (none/HEP/PUL/LYM/PER)	304/6/0/1/2
TNM stage (0/I/II/III/IV)	29/115/77/83/9
Complication (CD grade) (none/I/II/III/IV/V)	249/23/23/16/2/0
GNRI	99.0 (62.2–122.6)

CRC = colorectal cancer, BMI = body mass index, ALB = serum albumin, WBC = white blood cell, CRP = C-reactive protein, CEA = carcinoembryonic antigen, CA19–9 = carbohydrate antigen 19–9, tub1 = well differentiated adenocarcinoma, tub2 = moderately differentiated adenocarcinoma, por = poorly differentiated adenocarcinoma, pap = papillary adenocarcinoma, muc = mucinous adenocarcinoma, HEP = liver, PUL = pulmonary, LYM = extra-regional lymph node, PER = peritoneal, TNM = tumor-node-metastasis, CD = Clavien-Dindo, GNRI = geriatric nutritional risk index, Asterisk values indicate median (range).

Clinicopathological factors such as age, sex, body mass index (BMI), serum albumin level (ALB), white blood cells, C-reactive protein (CRP), carcinoembryonic antigen (CEA), carbohydrate antigen 19-9 (CA19-9), primary tumor location, distant metastases, pathological findings, and postoperative complications were collected from patients’ medical records. Clinicopathological factors were classified according to the eighth edition of the Union for International Cancer Control (UICC) tumor-node-metastasis (TNM) classification^25^. Preoperative blood samples, height, and weight data were obtained within 7 days before surgery. Postoperative complications were classified according to the Clavien-Dindo (CD) grade^26^. In the present study, we examined those of CD grade ≥II^27^.

After surgery, all patients were followed up according to the Japanese guidelines^28^. They were regularly examined using tumor markers, such as CEA and CA19-9, and screened using computed tomography every 3–6 months and colonoscopy every 1–2 years.

### Nutritional assessment by GNRI

The GNRI is a simple and objective screening tool for elderly patients’ nutrition-related risk calculated using ALB, present body weight (PBW), and ideal body weight (IBW). IBW in this study was calculated as follows: IBW = height^2^ (m) × 22. The GNRI formula is: GNRI = 1.487 × ALB (g/L) + 41.7 × PBW/IBW (kg)^17^.

### Statistical analysis

Continuous variables were expressed as means ± standard deviation (SD) values. Differences between the classified GNRI groups and clinicopathological factors were analysed using chi-squared test or Fisher’s exact test. The relationships between GNRI and each complication were also analysed by the same tests. Continuous variables with parametric distribution were analysed by Student’s t-test or analysis of variance (ANOVA). Overall survival (OS) curves were plotted using the Kaplan–Meier method and compared using the generalised log-rank test. Univariate and multivariate analyses were performed using a logistic regression model to identify independent risk factors for postoperative complications and using a Cox proportional hazards regression model for OS. Receiver operating characteristic (ROC) curve analysis was used to predict the optimal cut-off value of GNRI for OS^29^. In this study, we used the patients who were followed for at least one year as evaluable for the prognostic outcome to perform the ROC analysis. Then, the value was provided based on Youden’s index^30^. Two-sided *P* < 0.05 was considered to denote statistical significance. All statistical analyses were performed using JMP software version 13 (SAS Institute Inc., Cary, NC, USA).

### Compliance with ethical review

This study was performed in accordance with the principles of Declaration of Helsinki. This study was approved by the Institutional Review Boards of Osaka University and Osaka International Cancer Institute, and informed consent was obtained from all patients according to the guideline.

## Results

### Patient characteristics

Two hundred one (64.2%) males and 112 (35.8%) females were included in this study. Characteristics of all patients are listed in Table 1. The median age was 73 years (range, 65–94 years). There were 29 (9.3%) patients with stage 0, 115 (36.7%) patients with stage I, 77 (24.6%) patients with stage II, 83 (26.5%) patients with stage III, and 9 (2.9%) patients with stage IV. The stage IV cases included liver metastasis (6 cases), extra-regional lymph node metastasis (1 case), and peritoneal dissemination (2 cases). Sixty-four (20.4%) patients had postoperative complications and 41 (13.1%) patients had those of CD grade ≥II.

### Distribution and classification of GNRI

**Figure 1:**
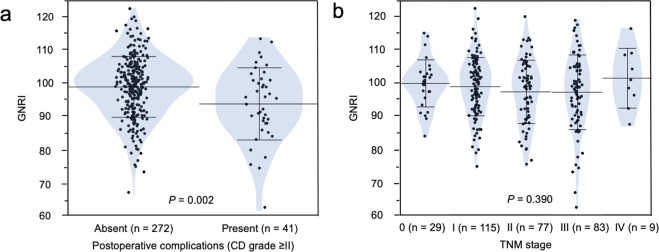
Distribution of GNRI according to (**a**) postoperative complications (Clavien-Dindo grade ≥II) and (**b**) TNM stages. (**a**) GNRI is significantly lower in patients with postoperative complications than in those without them (*P* = 0.002). (**b**) GNRI is not significantly different among TNM stages (*P* = 0.390).

The mean preoperative GNRI in 313 patients with CRC was 98.2 ± 9.6. Differences in the distribution of preoperative GNRI according to postoperative complications (CD grade ≥II) and TNM stages are shown in Fig. 1. The mean GNRI was 98.9 ± 9.2 in patients who had postoperative complications and 93.8 ± 11.0 in those without complications. There was a significant difference in preoperative GNRI between the two groups (*P* = 0.002) (Fig. 1a). The mean GNRI was 99.9 ± 7.1 in stage 0, 98.9 ± 8.8 in stage I, 97.3 ± 9.6 in stage II, 97.2 ± 11.3 in stage III, and 101.4 ± 9.1 in stage IV. There were no significant differences in preoperative GNRI among these stages (*P* = 0.390) (Fig. 1b).

**Figure 2:**
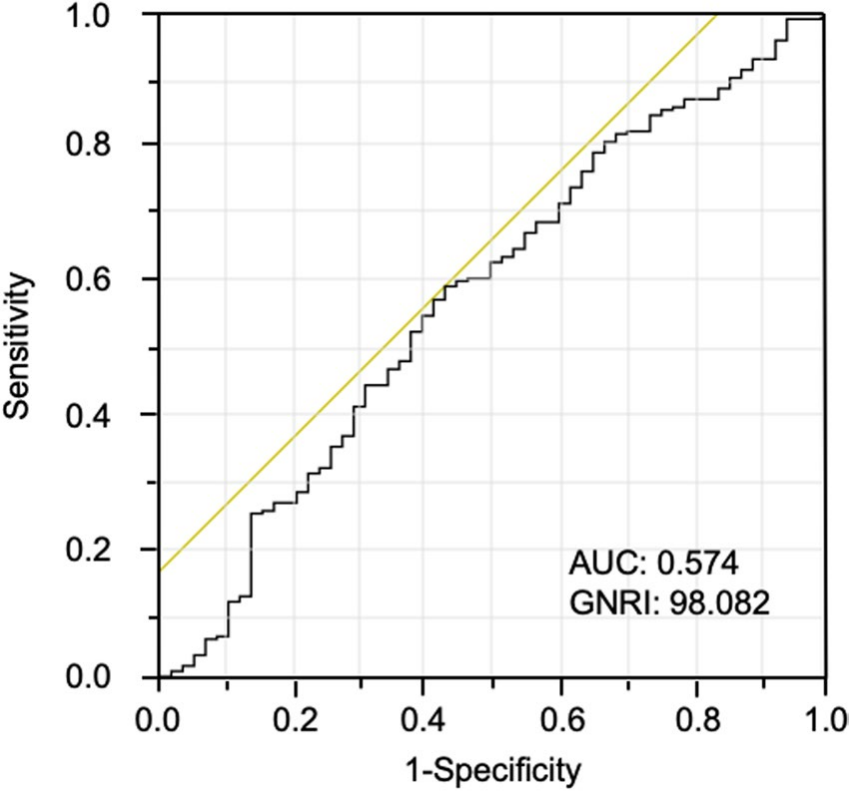
Receiver operating characteristic (ROC) curve analysis of GNRI for overall survival in elderly patients with colorectal cancer. The ROC curve shows that the optimal cut-off value of GNRI is 98.082. Area under the curve for GNRI is 0.574. The sensitivity is 0.591, and the specificity is 0.569.

A previous study showed that a good sensitivity for risk prediction was found only for a GNRI cut-off value of 98^31^. ROC curve analysis for OS also showed that the optimal cut-off value of GNRI was 98.082 (area under the curve = 0.574, sensitivity = 0.591, and specificity = 0.569) (Fig. 2).

According to previous studies^23,24,31^ and the ROC analysis, we classified patients more simply into an all-risk GNRI (≤98) group (137 patients, 43.8%) and a no-risk GNRI (>98) group (176 patients, 56.2%), instead of the four classifications of Bouillanne *et al*.^17^. The relationship between GNRI status and clinicopathological factors in all patients is shown in Table 2. Between the all- and no-risk GNRI groups, there were no significant differences in age, white blood cells, preoperative CEA, preoperative CA19-9, tumor location, degree of differentiation, depth of tumor invasion, lymph node metastasis, lymphatic vessel invasion, venous invasion, distant metastasis, or TNM stage. However, there were significant differences in sex, BMI, ALB, CRP, and postoperative complications (CD grade ≥II) between the two groups.

**Table 2:** The relationship between GNRI status and clinicopathological factors in the elderly patients with CRC.

Variables	GNRI		
	All-risk ≤ 98 (n = 137)	No-risk > 98 (n = 176)	*P*-value
Age (≥73/<73)	83/54	88/88	0.062
Sex (male/female)	77/60	124/52	0.009*
BMI (≥22/<22)	30/107	139/37	<0.001*
ALB (≥3.5/<3.5)	75/62	169/7	<0.001*
WBC (≥10000/<10000)	4/133	2/174	0.254
CRP (≥1/<1)	20/117	11/165	0.014*
Preoperative CEA (≥5/<5)	45/92	51/125	0.462
Preoperative CA19–9 (≥38/<38)	22/115	17/159	0.090
Tumor location (colon/rectum)	102/35	137/39	0.485
Degree of differentiation (tub1, tub2/por, pap, muc)	127/10	161/15	0.691
Depth of tumor invasion (Tis, T1, 2/T3, 4)	71/66	89/87	0.825
Lymph node metastasis (present/absent)	41/96	47/129	0.530
Lymphatic vessel invasion (present/absent)	90/47	106/70	0.321
Venous invasion (present/absent)	35/102	40/136	0.563
Distant metastasis (present/absent)	3/134	6/170	0.517
TNM stage (0-II/III, IV)	95/42	126/50	0.665
Complication (CD grade ≥II) (present/absent)	25/112	16/160	0.018*

GNRI = geriatric nutritional risk index, CRC = colorectal cancer, BMI = body mass index, ALB = serum albumin, WBC = white blood cell, CRP = C-reactive protein, CEA = carcinoembryonic antigen, CA19–9 = carbohydrate antigen 19–9, tub1 = well differentiated adenocarcinoma, tub2 = moderately differentiated adenocarcinoma, por = poorly differentiated adenocarcinoma, pap = papillary adenocarcinoma, muc = mucinous adenocarcinoma, TNM = tumor-node-metastasis, CD = Clavien-Dindo, Asterisk values indicate P-values < 0.05.

### Postoperative complications (CD grade ≥ II)

A total of 41 patients had postoperative complications defined CD grade ≥II. These were surgical site infection (11 cases), ileus (8 cases), anastomotic leakage (7 cases), intra-abdominal abscess (5 cases), colitis (4 cases), pneumonia (3 cases), and urinary infection (3 cases). More patients had postoperative complications in the all-risk GNRI group (18.2%) than in the no-risk GNRI group (9.1%) (*P* = 0.018). The relationship between GNRI status and each complication was examined, and surgical site infection occurrence was higher in the all-risk GNRI group than in the no-risk GNRI group (*P* = 0.008) (Table 3).

**Table 3:** The relationship between GNRI status and postoperative complications (CD grade ≥II) in the elderly patients with CRC.

Variables	Total (n = 313) (%)	GNRI		
		All-risk ≤ 98 (n = 137)	No-risk > 98 (n = 176)	*P-*value
All	41 (13.1)	25	16	0.018*
Surgical site infection	11 (3.5)	9	2	0.008*
Ileus	8 (2.6)	4	4	0.720
Leakage	7 (2.2)	3	4	0.961
Intra-abdominal abscess	5 (1.6)	3	2	0.463
Colitis	4 (1.3)	3	1	0.202
Pneumonia	3 (1.0)	1	2	0.711
Urinary infection	3 (1.0)	2	1	0.423

GNRI = geriatric nutritional risk index, CD = Clavien-Dindo, CRC = colorectal cancer, Asterisk values indicate P-values < 0.05.

Univariate and multivariate analyses of clinicopathological factors for postoperative complications (CD grade ≥II) are shown in Table 4. According to the univariate analysis, high CRP (*P* = 0.032), tumor location (rectum) (*P* = 0.005), and low GNRI (*P* = 0.019) were significantly correlated with the complications. The multivariate analysis showed that tumor location (rectum) (*P* = 0.005) and low GNRI (*P* = 0.048) were independent risk factors for postoperative complications.

**Table 4:** The univariate and multivariate analyses of predictors for postoperative complications (CD grade ≥II).

Variables	Univariate			Multivariate		
	RR	95%CI	*P*-value	RR	95%CI	*P*-value
Age (≥73/<73)	1.518	0.770–2.993	0.228			
Sex (male/female)	1.233	0.610–2.489	0.560			
BMI (≥22/<22)	0.880	0.456–1.697	0.702			
WBC (≥10000/<10000)	3.436	0.609–19.386	0.162			
CRP (≥1/<1)	2.625	1.086–6.344	0.032*	2.471	0.980–6.231	0.055
Preoperative CEA (≥5/<5)	1.730	0.882–3.396	0.111			
Preoperative CA19–9 (≥38/<38)	1.544	0.632–3.772	0.340			
Tumor location (rectum/colon)	2.672	1.345–5.308	0.005*	2.741	1.356–5.539	0.005*
Degree of differentiation (por, pap, muc/tub1, tub2)	1.292	0.420–3.974	0.655			
Depth of tumor invasion (T3, 4/Tis, T1, 2)	1.758	0.898–3.439	0.100			
Lymph node metastasis (present/absent)	1.067	0.518–2.199	0.860			
Lymphatic vessel invasion (present/absent)	1.333	0.661–2.690	0.422			
Venous invasion (present/absent)	1.794	0.886–3.632	0.105			
Distant metastasis (present/absent)	0.825	0.100–6.773	0.858			
GNRI (≤98/>98)	2.232	1.140–4.372	0.019*	2.001	1.002–3.999	0.048*

CD = Clavien-Dindo, RR = risk ratio, CI = confidence interval, BMI = body mass index, WBC = white blood cell, CRP = C-reactive protein, CEA = carcinoembryonic antigen, CA19–9 = carbohydrate antigen 19-9, por = poorly differentiated adenocarcinoma, pap = papillary adenocarcinoma, muc = mucinous adenocarcinoma, tub1 = well differentiated adenocarcinoma, tub2 = moderately differentiated adenocarcinoma, GNRI = geriatric nutritional risk index, Asterisk values indicate P-values < 0.05.

### Survival analysis and risk factors for mortality

**Figure 3:**
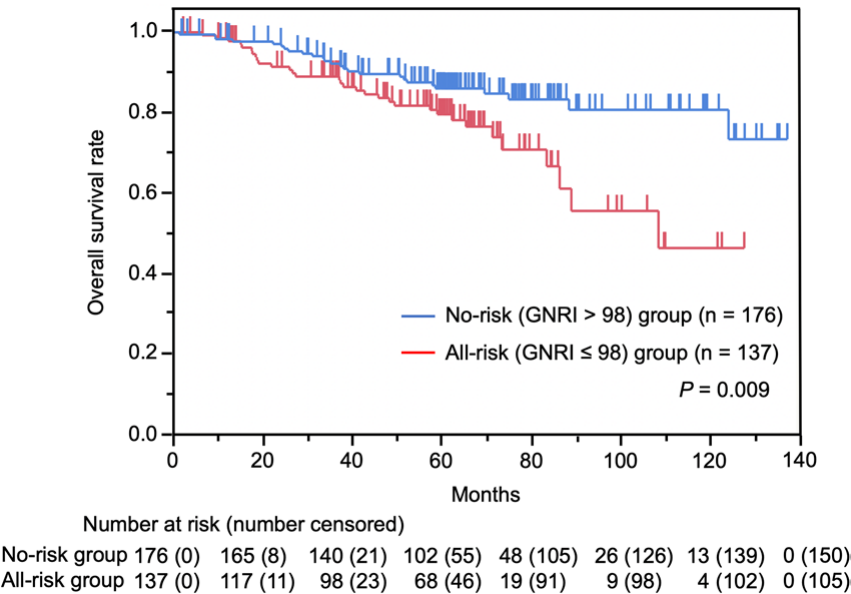
Kaplan-Meier analysis of overall survival according to GNRI. Overall survival rate is significantly worse in the all-risk GNRI (≤98) group than in the no-risk GNRI (>98) group (*P* = 0.009).

The median follow-up was 60.5 months (range, 1–137 months). Thirty-two death events and 105 censoring cases were recorded in the all-risk GNRI group, and 26 death events and 150 censoring cases were recorded in the no-risk GNRI group. OS rate was significantly worse in the all-risk GNRI group than in the no-risk GNRI group (*P* = 0.009) (Fig. 3). The 3- and 5-year OS rates in the all-risk GNRI group were 89.0% and 79.6%, and those in the no-risk GNRI group were 92.2% and 86.0%, respectively.

The univariate and multivariate analyses of clinicopathological factors for OS are shown in Table 5. According to the univariate analysis, sex (male) (*P* < 0.001), high preoperative CEA (*P* < 0.001), high preoperative CA19-9 (*P* < 0.001), depth of tumor invasion (T3, 4) (*P* < 0.001), lymph node metastasis (*P* < 0.001), lymphatic vessel invasion (*P* < 0.001), venous invasion (*P* < 0.001), distant metastasis (*P* < 0.001), and low GNRI (*P* = 0.010) were significantly correlated with OS. The multivariate analysis showed that sex (male) (*P* < 0.001), high preoperative CEA (*P* = 0.044), lymph node metastasis (*P* = 0.025), distant metastasis (*P* = 0.030), and low GNRI (*P* = 0.001) were independent prognostic risk factors for OS.

**Table 5:** The univariate and multivariate analyses of prognostic factors for overall survival.

Variables	Univariate			Multivariate		
	HR	95%CI	*P*-value	HR	95%CI	*P*-value
Age (≥73/<73)	1.627	0.957–2.851	0.073			
Sex (male/female)	2.992	1.545–6.519	<0.001*	3.668	1.850–8.137	<0.001*
BMI (≥22/<22)	1.224	0.727–2.100	0.451			
Preoperative CEA (≥5/<5)	2.446	1.454–4.102	<0.001*	1.875	1.018–3.416	0.044*
Preoperative CA19–9 (≥38/<38)	3.387	1.817–5.974	<0.001*	1.963	0.953–3.806	0.067
Tumor location (rectum/colon)	1.217	0.654–2.140	0.520			
Degree of differentiation (por, pap, muc/tub1, tub2)	1.581	0.607–3.404	0.318			
Depth of tumor invasion (T3, 4/Tis, T1, 2)	3.112	1.786–5.700	<0.001*	1.282	0.624–2.749	0.506
Lymph node metastasis (present/absent)	3.036	1.808–5.097	<0.001*	1.976	1.089–3.624	0.025*
Lymphatic vessel invasion (present/absent)	2.963	1.564–6.217	<0.001*	1.062	0.463–2.552	0.890
Venous invasion (present/absent)	3.371	1.983–5.658	<0.001*	1.844	0.997–3.385	0.051
Distant metastasis (present/absent)	8.131	3.303–17.262	<0.001*	3.055	1.122–7.507	0.030*
GNRI (≤98/>98)	1.988	1.179–3.384	0.010*	2.429	1.414–4.230	0.001*

HR = hazard ratio, CI = confidence interval, BMI = body mass index, CEA = carcinoembryonic antigen, CA19–9 = carbohydrate antigen 19–9, por = poorly differentiated adenocarcinoma, pap = papillary adenocarcinoma, muc = mucinous adenocarcinoma, tub1 = well differentiated adenocarcinoma, tub2 = moderately differentiated adenocarcinoma, GNRI = geriatric nutritional risk index, Asterisk values indicate *P*-values < 0.05.

### Analyses of the complications and prognosis in the other dataset based on GNRI

To verify whether GNRI could be used for the prediction, we performed the other center study using the patient data in Osaka International Cancer Institute. Characteristics of all the patients in the other dataset are listed in Supplementary Table 1. The median age was 72 years (range, 65–88 years). Fifty-three (24.3%) patients had postoperative complications of CD grade ≥II. The mean preoperative GNRI in the patients was 101.9 ± 9.2.

The univariate and multivariate analyses for the complications in the other center study are shown in Supplementary Table 2. According to the univariate analysis, tumor location (rectum) (*P* = 0.001), venous invasion (*P* = 0.047), and low GNRI (*P* < 0.0001) were significantly related to the complications. The multivariate analysis showed that tumor location (rectum) (*P* = 0.001) and low GNRI (*P* < 0.001) were independent risk factors for the complications.

Furthermore, Kaplan-Meyer curve analysis in the other center study also showed that OS rate was significantly worse in the all-risk GNRI group than in the no-risk GNRI group (*P* = 0.002) (Supplementary Fig. 1). The 3- and 5-year OS rates in the all-risk GNRI group were 83.7% and 77.6%, and those in the no-risk GNRI group were 96.7% and 91.1%, respectively. The univariate and multivariate analyses for OS are shown in Supplementary Table 3. According to the univariate analysis, lymph node metastasis (*P* = 0.004), distant metastasis (*P* = 0.001), and low GNRI (*P* = 0.005) were significantly related to OS. The multivariate analysis also showed that lymph node metastasis (*P* = 0.035), distant metastasis (*P* = 0.042), and low GNRI (*P* = 0.048) were independent prognostic risk factors for OS.

## Discussion

Our results showed that GNRI was associated with increased postoperative complications and poor prognosis of CRC in elderly patients. Malnutrition has been found to be an important risk factor for postoperative morbidity and mortality in malignant tumors^32,33^. The Nutritional Risk Index (NRI), calculated by ALB, PBW, and usual body weight, was proposed by Buzby *et al*. to evaluate the association between nutrition and postoperative complications^34,35^. However, the NRI is often difficult to use in elderly patients^36^ because half of them do not remember their own usual body weight^37^. Thus, Bouillanne *et al*. replaced usual body weight with IBW in the formula of NRI and developed a simple screening tool specific for elderly patients to predict nutrition-related risk of morbidity and mortality^17^. GNRI was developed in the population of which elderly patients aged ≥65 years were admitted into a geriatric rehabilitation care hospital due to rehabilitation after fractures, neurologic diseases, cardiovascular diseases, and postinfectious diseases and also reported to be significantly correlated with ALB, prealbumin, weight, and BMI^17^.

There are several methods for assessing nutritional status, such as BMI, prognostic nutritional index, skeletal muscle mass index, and subjective global assessment. While these measures are relevant for the prognosis of cancer^38–41^, optimal cut-off values remain to be elucidated. Additionally, subjective global assessment is based on many subjective factors, and expert knowledge is required to use it^41^.

In contrast, the advantage of GNRI is that it is an objective and easily available predicting tool. The classification value of GNRI has already been proposed^17^. Moreover, this index is calculated using ALB, height, and body weight, which are usually measured on admission.

Previously, GNRI was considered as a prognostic predictor for length of stay in hospital^31^ and chronic diseases in elderly patients, such as those with heart failure^18^ or chronic obstructive pulmonary disease^19^, or those undergoing haemodialysis^20^. Recently, GNRI has been reported to be useful as a predictor for morbidity and mortality in patients with cancer. Li *et al*. reported that lower GNRI value was associated with severe postoperative complications, including liver failure, and poor OS in elderly patients with hepatocellular carcinoma^21^. Kushiyama *et al*. suggested that GNRI < 92 was a risk factor for postoperative complications in elderly patients with gastric cancer^22^. Bo *et al*. indicated that GNRI ≤ 98 could be an indicator of poor survival in elderly patients with oesophageal cancer treated with radiotherapy^23^. Miyake *et al*. also reported that GNRI could be a prognostic predictor in elderly patients with non-metastatic renal cell carcinoma, and those with GNRI ≤ 98 had significantly worse cancer-specific survival (CSS) than those with GNRI > 98^24^.

Some reports used the modified GNRI classification according to the complications^22,42^, OS^23^, CSS^24^ and length of hospital stay^31^, and some reports used the four-group classification proposed by Bouillanne *et al*.^21,43^. In this study, we divided the patients more simply into two groups by the GNRI value 98 based on the ROC analysis and these previous studies^23,24^. Cereda *et al*. also suggested that only a GNRI cut-off value of 98 had good sensitivity for risk prediction^31^. Our results showed that GNRI was related to the complications and prognosis of CRC, and it was considered that our classification was appropriate.

Postoperative complications after CRC resection have been reported to be associated with poor oncologic outcomes, even if they are mild or moderate (CD grade II)^27^. For this reason, we considered not only severe complications (CD grade ≥III) but all complications of CD grade ≥II in the present study.

To the best of our knowledge, this is the first study to investigate the relationship between GNRI and outcomes in elderly patients with CRC. Our study demonstrated that low preoperative GNRI (≤98) was correlated with increased postoperative complications (CD grade ≥II) and worse OS compared with high GNRI (>98) and that low GNRI was an independent risk factor for morbidity and mortality. In addition, although we examined the relationship between GNRI and TNM stages, no significant correlation between them was found. Therefore, we considered that GNRI was also an independent prognostic factor that did not depend on TNM stage.

Several studies have suggested that preoperative nutritional status is an independent risk factor for anastomotic leakage and wound infection in patients with CRC^44,45^. Our study also showed that GNRI was a significant risk factor for wound infection, but it was not a risk factor for anastomotic leakage. Our result obtained for anastomotic leakage might be due to the small number of cases, and these may have been more influenced by tumor location and surgical procedure. Some studies also showed that enhanced recovery after surgery protocol was associated with decreased postoperative complications^46^ and improved survival in CRC^47^. Appropriate management of nutritional status before and after surgery may be important to improve surgical risk and prognosis.

Low ALB is correlated with poor prognosis of cancer^48^. ALB is a known indicator of nutritional status^49^, and malnutrition impairs various functions, such as immunity, digestive tract function, and wound healing^50^. Deficiency of these functions increases the risk of infection and postoperative complications^51,52^, and an immunosuppressed condition leads to inadequate anti-tumor immunological reaction^53,54^. Furthermore, ALB is also influenced by inflammation^49^, and systemic inflammation is associated with poor prognosis of cancer^55^. On the other hand, the PBW/IBW ratio used in GNRI, which replaces the PBW/usual body weight ratio indicating weight loss, might be interpreted as reflecting the degree of frailty and cachexia associated with poor prognosis in elderly patients^56^. Thus, the GNRI, which combines factors of ALB and body weight, may predict nutrition-related risk better than ALB alone.

There are some limitations to our study. First, this study was a retrospective study evaluated only a small number of patients and institutes, and also affected by some selection and information bias. Prospective multicenter studies should be performed. Second, there is no single definition of elderly patients. While we defined elderly patients as those aged ≥ 65 years in the present study, the life span has extended and the number of patients aged>80 years has been increasing. Similar analyses may also have to be performed in patients aged >80 years. Third, our study did not assess the influence of smoking behavior because of lack of the information. Smoking is well known as a risk factor of malnutrition, postoperative complications, and poor cancer prognosis^57–59^. In contrast, there is no consensus on the association between smoking and BMI or body weight^59,60^. How smoking actually influences on GNRI status and our findings is not clear, and further research including smoking status is necessary to make it more meaningful and accurate.

In conclusion, our study demonstrated that low preoperative GNRI value (≤98) was associated with increased postoperative complications and poor prognosis in patients with CRC aged ≥65 years after curative surgery. Preoperative GNRI can be a useful tool to identify high-risk population of morbidity and mortality in elderly patients with CRC.

## Supplementary Information

Supplementary Information.

Supplementary Information.

## Data Availability

The dataset used and analysed in the present study is available from the corresponding author on reasonable request.

## References

[1] Bray F (2018). Global cancer statistics 2018: GLOBOCAN estimates of incidence and mortality worldwide for 36 cancers in 185 countries. CA Cancer J Clin..

[2] Siegel RL, Miller KD, Jemal A (2019). Cancer statistics, 2019. CA Cancer J Clin..

[3] International Agency for Research on Cancer WHO. Cancer Today - IARC. Data visualization tools for exploring the global cancer burden in 2018, https://gco.iarc.fr/today.

[4] Etzioni DA, Liu JH, Maggard MA, Ko CY (2003). The aging population and its impact on the surgery workforce. Ann Surg..

[5] Karakoc D (2016). Surgery of the Elderly Patient. Int Surg..

[6] DeSantis CE (2019). Cancer statistics for adults aged 85 years and older, 2019. CA Cancer J Clin..

[7] Endo S (2013). Prognosis of gastric carcinoma patients aged 85 years or older who underwent surgery or who received best supportive care only. Int J Clin Oncol..

[8] Yoshida M (2017). Laparoscopy- assisted distal gastrectomy is feasible also for elderly patients aged 80 years and over: effectiveness and long-term prognosis. Surg Endosc..

[9] Chen CC, Schilling LS, Lyder CH (2001). A concept analysis of malnutrition in the elderly. J Adv Nurs..

[10] DiMaria-Ghalili RA, Amella E (2005). Nutrition in older adults. Am J Nurs..

[11] Norman K, Pichard C, Lochs H, Pirlich M (2008). Prognostic impact of disease-related malnutrition. Clin Nutr..

[12] Caccialanza R, Cereda E, Klersy C (2011). Malnutrition, age and inhospital mortality. CMAJ..

[13] Yasui A (2007). Activities of daily living and quality of life of elderly patients after elective surgery for gastric and colorectal cancers. Ann Surg..

[14] Van Cutsem E, Arends J (2005). The causes and consequences of cancer-associated malnutrition. Eur J Oncol Nurs..

[15] Abd-El-Gawad WM, Abou-Hashem RM, El Maraghy MO, Amin GE (2014). The validity of Geriatric Nutrition Risk Index: Simple tool for prediction of nutritional-related complication of hospitalized elderly patients. Comparison with Mini Nutritional Assessment. Clin Nutr..

[16] Cereda E (2013). Nutritional risk, functional status and mortality in newly institutionalised elderly. Br J Nutr..

[17] Bouillanne O (2005). Geriatric Nutritional Risk Index: a new index for evaluating at-risk elderly medical patients. Am J Clin Nutr..

[18] Kaneko H (2015). Geriatric nutritional risk index in hospitalized heart failure patients. Int J Cardiol..

[19] Matsumura T (2015). Comparison of Geriatric Nutritional Risk Index scores on physical performance among elderly patients with chronic obstructive pulmonary disease. Heart Lung..

[20] Komatsu M, Okazaki M, Tsuchiya K, Kawaguchi H, Nitta K (2015). Geriatric Nutritional Risk Index Is a Simple Predictor of Mortality in Chronic Hemodialysis Patients. Blood Purif..

[21] Li L (2018). Geriatric nutritional risk index predicts prognosis after hepatectomy in elderly patients with hepatitis B virus-related hepatocellular carcinoma. Sci Rep..

[22] Kushiyama S (2018). The Preoperative Geriatric Nutritional Risk Index Predicts Postoperative Complications in Elderly Patients with Gastric Cancer Undergoing Gastrectomy. In Vivo..

[23] Bo Y (2016). The Geriatric Nutritional Risk Index Predicts Survival in Elderly Esophageal Squamous Cell Carcinoma Patients with Radiotherapy. PLoS One..

[24] Miyake H, Tei H, Fujisawa M (2017). Geriatric Nutrition Risk Index is an Important Predictor of Cancer-Specific Survival, but not Recurrence-Free Survival, in Patients Undergoing Surgical Resection for Non-Metastatic Renal Cell Carcinoma. Curr Urol..

[25] Brierley, J. D., Gospodarowicz, M. K. & Wittekind, C. TNM classification of malignant tumours, eighth edition. 73–77 (Wiley-Blackwell, 2017).

[26] Dindo D, Demartines N, Clavien PA (2004). Classification of surgical complications: a new proposal with evaluation in a cohort of 6336 patients and results of a survey. Ann Surg..

[27] Duraes LC (2018). The Relationship Between Clavien-Dindo Morbidity Classification and Oncologic Outcomes After Colorectal Cancer Resection. Ann Surg Oncol..

[28] Watanabe T (2012). Japanese Society for Cancer of the Colon and Rectum (JSCCR) guidelines 2010 for the treatment of colorectal cancer. Int J Clin Oncol..

[29] Iseki Y (2015). Impact of the preoperative controlling nutritional status (CONUT) score on the survival after curative surgery for colorectal cancer. PLoS One..

[30] Youden WJ (1950). Index for rating diagnostic tests. Cancer..

[31] Cereda E (2015). The Geriatric Nutritional Risk Index predicts hospital length of stay and in-hospital weight loss in elderly patients. Clin Nutr..

[32] Schwegler I (2010). Nutritional risk is a clinical predictor of postoperative mortality and morbidity in surgery for colorectal cancer. Br J Surg..

[33] Kanda M (2011). Nutritional predictors of post- operative outcome in pancreatic cancer. Br J Surg..

[34] Buzby GP (1988). A randomized clinical trial of total parenteral nutrition in malnourished surgical patients: the rationale and impact of previous clinical trials and pilot study on protocol design. Am J Clin Nutr..

[35] Buzby GP (1988). Study protocol: a randomized clinical trial of total parenteral nutrition in malnourished surgical patients. Am J Clin Nutr..

[36] Kuczmarski MF, Kuczmarski RJ, Najjar M (2001). Effects of age on validity of self-reported height, weight, and body mass index: findings from the Third National Health and Nutrition Examination Survey, 1988–1994. J Am Diet Assoc..

[37] Robbins LJ (1989). Evaluation of weight loss in the elderly. Geriatrics..

[38] Adachi T (2016). Lower body mass index predicts worse cancer-specific prognosis in octogenarians with colorectal cancer. J Gastroenterol..

[39] Shibutani M (2015). The prognostic significance of the postoperative prognostic nutritional index in patients with colorectal cancer. BMC Cancer..

[40] Reisinger KW (2015). Loss of Skeletal Muscle Mass During Neoadjuvant Chemoradiotherapy Predicts Postoperative Mortality in Esophageal Cancer Surgery. Ann Surg Oncol..

[41] Gupta D (2005). Prognostic significance of Subjective Global Assessment (SGA) in advanced colorectal cancer. Eur J Clin Nutr..

[42] Cereda E, Pusani C, Limonta D, Vanotti A (2009). The ability of the Geriatric Nutritional Risk Index to assess the nutritional status and predict the outcome of home-care resident elderly: a comparison with the Mini Nutritional Assessment. Br J Nutr..

[43] Wang H, Hai S, Zhou Y, Liu P, Dong BR (2018). The Geriatric Nutritional Risk Index predicts mortality in nonagenarians and centenarians receiving home care. Asia Pac J Clin Nutr..

[44] Frasson M (2015). Risk factors for anastomotic leak after colon resection for cancer: Multivariate Analysis and nomogram from a multicentric, prospective, national study with 3193 patients. Ann Surg..

[45] Tanaka T (2017). Effect of Preoperative Nutritional Status on Surgical Site Infection in Colorectal Cancer Resection. Dig Surg..

[46] Eskicioglu C (2009). Enhanced Recovery after Surgery (ERAS) Programs for Patients Having Colorectal Surgery: A Meta-analysis of Randomized Trials. J Gastrointest Surg..

[47] Gustafsson UO (2016). Adherence to the ERAS protocol is Associated with 5-Year Survival After Colorectal Cancer Surgery: A Retrospective Cohort Study. World J Surg..

[48] Gupta D, Lis CG (2010). Pretreatment serum albumin as a predictor of cancer survival: a systematic review of the epidemiological literature. Nutr J..

[49] de Ulíbarri Pérez JI, Fernández G, Rodríguez Salvanés F, Díaz López AM (2014). Nutritional screening; control of clinical undernutrition with analytical parameters. Nutr Hosp..

[50] Saunders J, Smith T, Stroud M (2011). Malnutrition and undernutrition. Medicine (Baltimore)..

[51] Lesourd B, Mazari L (1999). Nutrition and immunity in the elderly. Proc Nutr Soc..

[52] Pae M, Meydani SN, Wu D (2012). The role of nutrition in enhancing immunity in aging. Aging Dis..

[53] Ray-Coquard I (2009). Lymphopenia as a prognostic factor for overall survival in advanced carcinomas, sarcomas, and lymphomas. Cancer Res..

[54] Mazur G (2006). TGF-beta1 gene polymorphisms influence the course of the disease in non-Hodgkin’s lymphoma patients. Cytokine..

[55] Diakos CI, Charles KA, McMillan DC, Clarke SJ (2014). Cancer-related inflammation and treatment effectiveness. Lancet Oncol..

[56] Fried LP (2001). Frailty in older adults: evidence for a phenotype. J Gerontol A Biol Sci Med Sci..

[57] Silva FR, de Oliveira MG, Souza AS, Figueroa JN, Santos CS (2015). Factors associated with malnutrition in hospitalized cancer patients: a croos-sectional study. Nutr J..

[58] McSorley ST, Watt DG, Horgan PG, McMillan DC (2016). Postoperative Systemic Inflammatory Response, Complication Severity, and Survival Following Surgery for Colorectal Cancer. Ann Surg Oncol..

[59] Jayasekara H (2018). Associations of alcohol intake, smoking, physical activity and obesity with survival following colorectal cancer diagnosis by stage, anatomic site and tumor molecular subtype. Int J Cancer..

[60] Carreras-Torres R (2018). Role of obesity in smoking behavior: Mendelian randomization study in UK Biobank. BMJ..

