# Supplementary material for: The Geriatric Nutritional Risk Index predicts postoperative complications and prognosis in elderly patients with colorectal cancer after curative surgery
Source: Sci Rep. 2020 Jul 1;10:10744. doi: 10.1038/s41598-020-67285-y (PMC7329855; doi:10.1038/s41598-020-67285-y)
Supplement: Supplementary file 1 — Supplementary Information. [file 41598_2020_67285_MOESM1_ESM.docx]

**Supplementary information**

**Title:**

The Geriatric Nutritional Risk Index predicts postoperative complications and prognosis in elderly patients with colorectal cancer after curative surgery

**Names of the authors:**

Masaru Sasaki^1^, Norikatsu Miyoshi^1,2*^, Shiki Fujino^1^, Takayuki Ogino^1^, Hidekazu Takahashi^1^, Mamoru Uemura^1^, Chu Matsuda^1^, Hirofumi Yamamoto^1^, Tsunekazu Mizushima^1^, Masaki Mori^3^, Yuichiro Doki^1^

**Institutional affiliations and the location of the institution:**

^1^Department of Gastroenterological Surgery, Osaka University Graduate School of Medicine, Suita, Japan

^2^Department of Innovative Oncology Research and Regenerative Medicine, Osaka International Cancer Institute, Osaka, Japan

^3^Department of Surgery and Science, Graduate School of Medical Sciences, Kyushu University, Fukuoka, Japan

*Correspondence and requests for materials should be addressed to N.M. ([nmiyoshi@gesurg.med.osaka-u.ac.jp](mailto:nmiyoshi@gesurg.med.osaka-u.ac.jp))

**Supplementary Figures**

**Supplementary Fig. 1:** **Kaplan-Meier analysis of overall survival according to GNRI in the other center study.** Overall survival rate is significantly worse in the all-risk GNRI (≤98) group than in the no-risk GNRI (>98) group (*P* = 0.002).


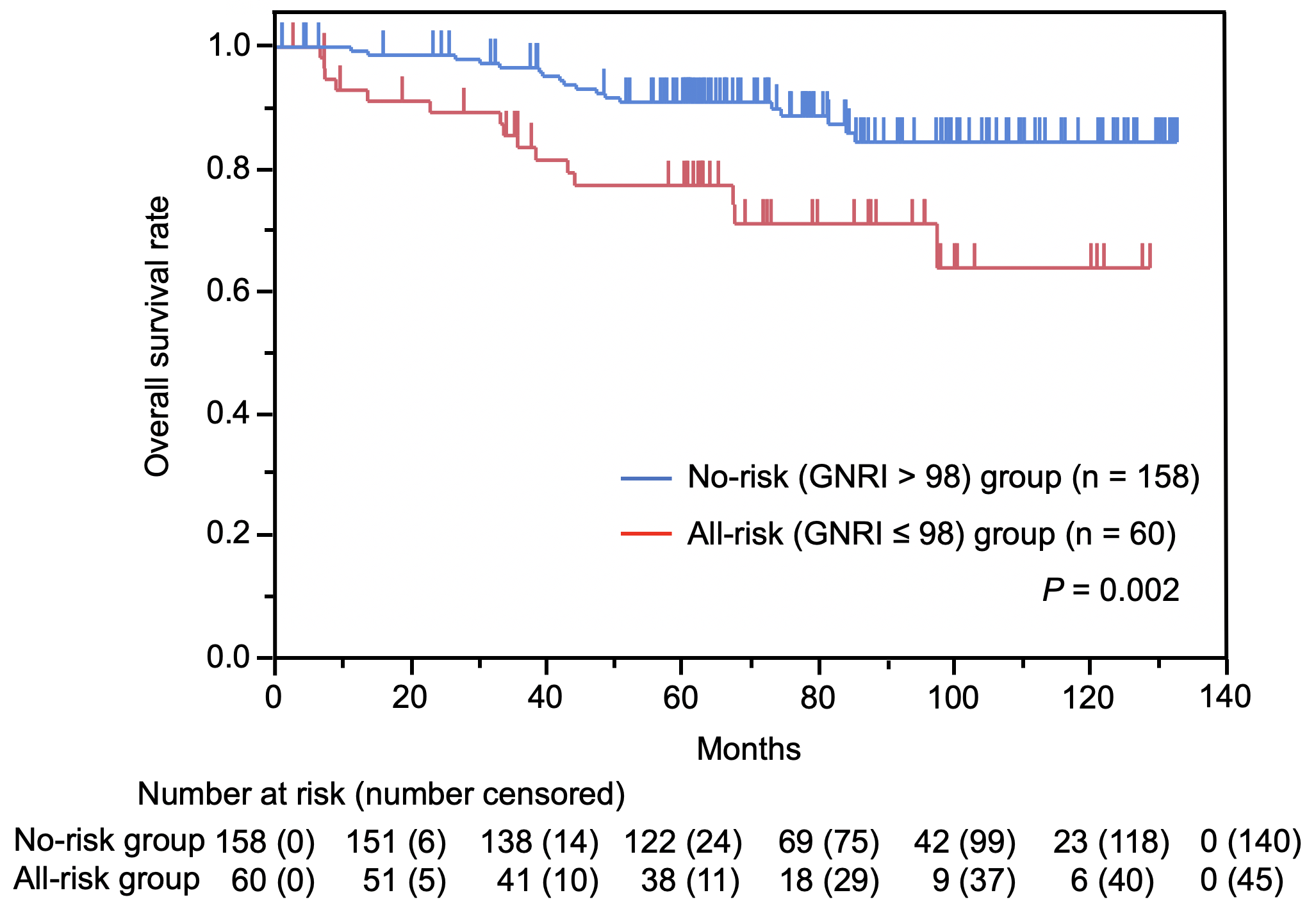


**Supplementary Tables**

**Supplementary Table 1: The characteristics of 218 patients with CRC in the other center study**

| **Variables** | **Total (n=218)** | **GNRI** | |
| --- | --- | --- | --- |
|  |  | **All-risk ≤98 (n=60)** | **No-risk >98 (n=158)** |
| Age (years)* | 72 (65-88) | 73 (65-88) | 71 (65-83) |
| Sex |  |  |  |
| male/female | 132/86 | 35/25 | 97/61 |
| BMI (kg/m^2^)* | 23.0 (16.2-33.8) | 20.6 (16.2-24.6) | 23.8 (18.6-33.8) |
| ALB (g/dl)* | 4.0 (2.5-4.7) | 3.5 (2.5-4.3) | 4.1 (3.2-4.7) |
| WBC (/μl)* | 5510 (2740-10980) | 5420 (2740-10980) | 5600 (3120-10770) |
| CRP (mg/dl)* | 0.05 (0.01-10.4) | 0.20 (0.01-9.24) | 0.03 (0.01-10.4) |
| Preoperative CEA (ng/ml)* | 3 (0.4-205) | 4 (0.4-63) | 3 (0.4-205) |
| Preoperative CA19-9 (U/ml)* | 10 (0-520) | 10 (0-344) | 11 (0-520) |
| Tumor location |  |  |  |
| colon/rectum | 148/70 | 41/19 | 107/51 |
| Degree of differentiation |  |  |  |
| tub1/tub2/por/pap/muc | 76/129/6/0/7 | 12/41/2/0/5 | 64/88/4/0/2 |
| Depth of tumor invasion |  |  |  |
| Tis/T1/T2/T3/T4 | 0/29/36/101/52 | 0/3/6/28/23 | 0/26/30/73/29 |
| Lymph node metastasis |  |  |  |
| N0/N1/N2 | 127/61/30 | 33/20/7 | 94/41/23 |
| Lymphatic vessel invasion |  |  |  |
| ly0/ly1/ly2/ly3 | 81/121/15/1 | 15/40/5/0 | 66/81/10/1 |
| Venous invasion |  |  |  |
| v0/v1/v2/v3 | 100/95/23/0 | 24/28/8/0 | 76/67/15/0 |
| Distant metastasis |  |  |  |
| none/HEP/PUL/LYM/PER | 207/7/1/2/1 | 54/3/0/2/1 | 153/4/1/0/0 |
| TNM stage |  |  |  |
| 0/I/II/III/IV | 0/53/72/82/11 | 0/9/24/21/6 | 0/44/48/61/5 |
| Complication (CD grade) |  |  |  |
| none/I/II/III/IV/V | 148/17/33/16/4/0 | 33/1/13/10/3/0 | 115/16/20/6/1/0 |

CRC = colorectal cancer, GNRI = geriatric nutritional risk index, BMI = body mass index, ALB = serum albumin, WBC = white blood cell, CRP = C-reactive protein, CEA = carcinoembryonic antigen, CA19-9 = carbohydrate antigen 19-9, tub1 = well differentiated adenocarcinoma, tub2 = moderately differentiated adenocarcinoma, por = poorly differentiated adenocarcinoma, pap = papillary adenocarcinoma, muc = mucinous adenocarcinoma, HEP = liver, PUL = pulmonary, LYM = extra-regional lymph node, PER = peritoneal, TNM = tumor-node-metastasis, CD = Clavien-Dindo, Asterisk values indicate median (range).

**Supplementary Table 2: The univariate and multivariate analyses of predictors for postoperative complications (CD grade ≥II) in the other center study**

|  | **Univariate** | | |  | **Multivariate** | | |
| --- | --- | --- | --- | --- | --- | --- | --- |
| **Variables** | **RR** | **95%CI** | ***P*-value** |  | **RR** | **95%CI** | ***P*-value** |
| Age (≥72/<72) | 1.572 | 0.840-2.939 | 0.157 |  |  |  |  |
| Sex (male/female) | 1.363 | 0.713-2.605 | 0.348 |  |  |  |  |
| BMI (≥22/<22) | 0.724 | 0.384-1.365 | 0.318 |  |  |  |  |
| WBC (≥10000/<10000) | 1.567 | 0.139-17.637 | 0.716 |  |  |  |  |
| CRP (≥1/<1) | 1.080 | 0.410-2.850 | 0.876 |  |  |  |  |
| Preoperative CEA (≥5/<5) | 1.307 | 0.697-2.450 | 0.404 |  |  |  |  |
| Preoperative CA19-9 (≥38/<38) | 1.064 | 0.429-2.642 | 0.893 |  |  |  |  |
| Tumor location (rectum/colon) | 2.946 | 1.552-5.593 | 0.001* |  | 3.210 | 1.619-6.363 | 0.001* |
| Degree of differentiation (por, pap, muc/tub1, tub2) | 0.245 | 0.031-1.932 | 0.182 |  |  |  |  |
| Depth of tumor invasion (T3, 4/Tis, T1, 2) | 1.416 | 0.698-2.870 | 0.335 |  |  |  |  |
| Lymph node metastasis (present/absent) | 1.013 | 0.541-1.898 | 0.968 |  |  |  |  |
| Lymphatic vessel invasion (present/absent) | 1.077 | 0.566-2.050 | 0.821 |  |  |  |  |
| Venous invasion (present/absent) | 1.921 | 1.008-3.662 | 0.047* |  | 1.740 | 0.872-3.470 | 0.116 |
| Distant metastasis (present/absent) | 2.760 | 0.807-9.443 | 0.106 |  |  |  |  |
| GNRI (≤98/>98) | 3.710 | 1.922-7.161 | <0.001* |  | 4.055 | 2.025-8.116 | <0.001* |

CD = Clavien-Dindo, RR = risk ratio, CI = confidence interval, BMI = body mass index, WBC = white blood cell, CRP = C-reactive protein, CEA = carcinoembryonic antigen, CA19-9 = carbohydrate antigen 19-9, por = poorly differentiated adenocarcinoma, pap = papillary adenocarcinoma, muc = mucinous adenocarcinoma, tub1 = well differentiated adenocarcinoma, tub2 = moderately differentiated adenocarcinoma, GNRI = geriatric nutritional risk index, Asterisk values indicate *P*-values < 0.05.

**Supplementary Table 3: The univariate and multivariate analyses of prognostic factors for overall survival in the other center study**

|  | **Univariate** | | |  | **Multivariate** | | |
| --- | --- | --- | --- | --- | --- | --- | --- |
| **Variables** | **HR** | **95%CI** | ***P*-value** |  | **HR** | **95%CI** | ***P*-value** |
| Age (≥72/<72) | 1.266 | 0.637-2.573 | 0.503 |  |  |  |  |
| Sex (male/female) | 1.170 | 0.575-2.321 | 0.658 |  |  |  |  |
| BMI (≥22/<22) | 0.569 | 0.286-1.136 | 0.109 |  |  |  |  |
| Preoperative CEA (≥5/<5) | 1.919 | 0.965-3.836 | 0.063 |  |  |  |  |
| Preoperative CA19-9 (≥38/<38) | 2.156 | 0.909-4.576 | 0.078 |  |  |  |  |
| Tumor location (rectum/colon) | 1.421 | 0.690-2.829 | 0.331 |  |  |  |  |
| Degree of differentiation (por, pap, muc/tub1, tub2) | 1.639 | 0.393-4.608 | 0.445 |  |  |  |  |
| Depth of tumor invasion (T3, 4/Tis, T1, 2) | 1.174 | 0.565-2.668 | 0.679 |  |  |  |  |
| Lymph node metastasis (present/absent) | 2.821 | 1.397-6.043 | 0.004* |  | 2.241 | 1.059-4.944 | 0.035* |
| Lymphatic vessel invasion (present/absent) | 1.499 | 0.720-3.413 | 0.289 |  |  |  |  |
| Venous invasion (present/absent) | 1.983 | 0.981-4.249 | 0.057 |  |  |  |  |
| Distant metastasis (present/absent) | 6.159 | 2.276-14.170 | 0.001* |  | 3.044 | 1.046-7.845 | 0.042* |
| GNRI (≤98/>98) | 2.769 | 1.374-5.500 | 0.005* |  | 2.130 | 1.005-4.400 | 0.048* |

HR = hazard ratio, CI = confidence interval, BMI = body mass index, CEA = carcinoembryonic antigen, CA19-9 = carbohydrate antigen 19-9, por = poorly differentiated adenocarcinoma, pap = papillary adenocarcinoma, muc = mucinous adenocarcinoma, tub1 = well differentiated adenocarcinoma, tub2 = moderately differentiated adenocarcinoma, GNRI = geriatric nutritional risk index, Asterisk values indicate *P*-values < 0.05.
