# Supplementary figures and images for: The Geriatric Nutritional Risk Index predicts postoperative complications and prognosis in elderly patients with colorectal cancer after curative surgery
Source: Sci Rep. 2020 Jul 1;10:10744. doi: 10.1038/s41598-020-67285-y (PMC7329855; doi:10.1038/s41598-020-67285-y)

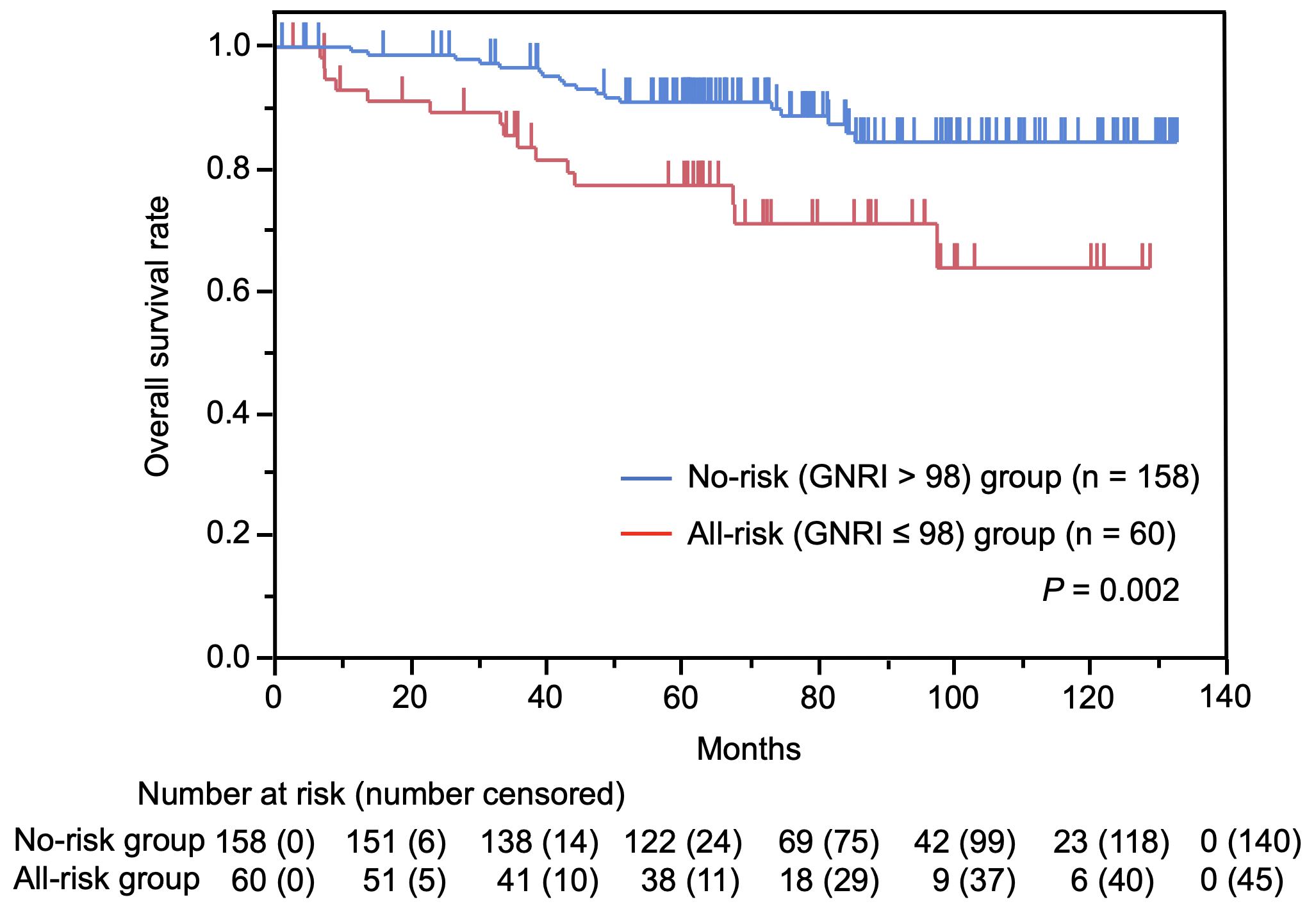

Supplement: Supplementary file 2 — Supplementary Information. [file 41598_2020_67285_MOESM2_ESM.png]
